# Development of Patient-Derived Preclinical Platform for Metastatic Pancreatic Cancer: PDOX and a Subsequent Organoid Model System Using Percutaneous Biopsy Samples

**DOI:** 10.3389/fonc.2019.00875

**Published:** 2019-09-13

**Authors:** Sun Il Choi, A-Ra Jeon, Min Kyeong Kim, Yu-Sun Lee, Ji Eun Im, Jung-Wook Koh, Sung-Sik Han, Sun-Young Kong, Kyong-Ah Yoon, Young-Hwan Koh, Ju Hee Lee, Woo Jin Lee, Sang-Jae Park, En Kyung Hong, Sang Myung Woo, Yun-Hee Kim

**Affiliations:** ^1^Division of Convergence Technology, Research Institute of National Cancer Center, Goyang, South Korea; ^2^Department of Life Science, Ewha Womans University, Seoul, South Korea; ^3^Division of Translational Science, Research Institute of National Cancer Center, Goyang, South Korea; ^4^Department of Biology, College of Education, Seoul National University, Seoul, South Korea; ^5^Center for Liver and Pancreatobiliary Cancer, National Cancer Center, Goyang, South Korea; ^6^Department of Laboratory Medicine, Center for Diagnostic Oncology, National Cancer Center, Goyang, South Korea; ^7^Department of Cancer Biomedical Science, The National Cancer Center Graduate School of Cancer Science and Policy, Goyang, South Korea; ^8^College of Veterinary Medicine, Konkuk University, Seoul, South Korea; ^9^Center for Diagnosic Oncology, National Cancer Center, Goyang, South Korea; ^10^Division of Tumor Immunology, National Cancer Center, Goyang, South Korea

**Keywords:** pancreatic ductal adenocarcinoma, preclinical cancer models, percutaneous liver biopsy, patient-derived orthotopic xenograft, organoid

## Abstract

Pancreatic ductal adenocarcinoma (PDAC) is the most lethal malignant tumor and more than 50% patients are diagnosed at metastatic stage. The preclinical model systems that reflect the genetic heterogeneity of metastatic tumors are urgently needed to guide optimal treatment. This study describes the development of patient-derived preclinical platform using very small sized-percutaneous liver gun biopsy (PLB) of metastatic pancreatic cancer, based on patient-derived xenograft (PDX)-mediated tissue amplification and subsequent organoid generation. To increase the success rate and shorten the tumor growth period, patient-derived orthotopic xenograft (PDOX) model was developed to directly implant threadlike PLB samples into the pancreas. The engraftment success rate of PDOX samples from 35 patients with metastatic PDAC was 47%, with these samples showing the potential to metastasize to distant organs, as in patients. The PDOX models retained the genetic alterations and histopathological features of the primary tumors. Tumor organoids were subsequently generated from first passage cancer cells isolated from F1 tumor tissue of PDOX that preserve the epithelial cancer characteristics and KRAS mutations of primary tumors. The response to gemcitabine of PDOX-derived organoids correlated with clinical outcomes in corresponding patients as well as PDOX models *in vivo*, suggesting that this PDOX-organoid system reflects clinical conditions. Collectively, these findings indicate that the proposed PDOX-organoid platform using PLB samples assessed both *in vitro* and *in vivo* could predict drug response under conditions closer to those found in actual patients, as well as enhancing understanding of the complexity of metastatic PDAC.

## Introduction

Despite advances in cancer research and therapeutic drug development, pancreatic ductal adenocarcinoma (PDAC) remains a highly lethal disease with a 5-year overall survival rate of <9% (1, 2). Surgical resection is the only curative treatment for PDAC, but fewer than 10% of patients have potentially resectable tumors at initial diagnosis. Approximately 80% of patients present with unresectable regional or systemic disease, excluding the possibility of curative therapy (3). Although preclinical studies have demonstrated the susceptibility of pancreatic cancer cells to cytotoxic and targeted chemotherapeutic agents, most primary PDACs are highly resistant to treatment and progress by an unknown mechanism. These findings suggest that current assay systems in drug development, including the use of highly passaged cancer cell lines, may not reflect actual patient status (4, 5). Although conventional cell models have substantially contributed to the current understanding of pancreatic cancer biology and treatment, a link connecting cell cultures and their clinical applications is absent (6).

Significant advances in pancreatic cancer can be made by developing preclinical models that recapitulate the native tumor environment and tumor heterogeneity. Patient-derived xenograft (PDX) models represent this missing link, as they enable the examination of tumor tissue in a native environment without significantly affecting the heterogeneity, genomics, and stromal architecture of these neoplasms (7). Abundant tissues from surgically resected early stage pancreatic tumors have been used to establish PDX models (8, 9). However, due to the high rate of non-resectable tumors at initial diagnosis and early distant metastasis after curative resection, most pancreatic cancer patient-derived samples of advanced pancreatic cancers can be obtained from core biopsies such as percutaneous liver biopsy (PLB). The liver is the most common site of pancreatic cancer metastasis. In most samples, the amount of recovered tumor sample is the major limiting factor for diagnosis and subsequent use in research. PDX samples implanted into the flanks of mice may fail to generate tumors or require long periods of time to form tumors. In addition, subcutaneous injection of PDX samples into mouse flanks may fail to generate systemic metastases as the key to the progression of primary PDACs. These models therefore cannot be enough to cover all stages and types of PDAC. Use of patient-derived orthotopic xenograft (PDOX) models derived from PLB samples may increase the success rate of PDX models and enable these tumors to metastasize.

Organoids have recently emerged as robust preclinical models of patient responses to drugs in the clinic (10). Tumor organoids have logistic, ethical, and economic advantages over PDX models in mice, being intermediate between cancer cell lines *in vitro* and xenografts *in vivo* (10–15). However, the rate of establishment of patient-derived organoids (PDOs) was found to correlate strongly with tumor cellularity in the biopsy samples. Specifically, PLB samples often fail to meet the tumor cellularity threshold for establishment of PDOs. Therefore, it is a major challenge to establish organoid-based assay systems that are needed for the efficient and high-throughput screening of drugs to treat PDACs.

Given the urgent need for better preclinical models of PDAC covering aspects of advanced pancreatic cancer, in the present work we sought to develop a PDOX model using PLB and a paired organoid platform based on PDX-mediated PLB sample amplification and subsequent organoid generation. We hypothesized that PDOX models would more effectively give rise to growing tumors and that they would rapidly differ from subcutaneous xenograft models, despite the very low cellularity of PLB samples. Moreover, PDOX models would be a better organoid source with sufficient neoplastic cellularity similar to surgical tissue and original characteristics of patient tumor. This study demonstrated that PDOX and a subsequent organoid model system using PLB were clinically relevant and reflected the pathological and molecular characteristics of the original tumor. Cross-validation of responses to drugs in both organoids and corresponding PDOX models may provide better evidence of drug responsiveness in patients with PDAC. This PDOX-organoid system for assessing metastatic PDAC may constitute an important preclinical model system with enhanced clinical relevance, augmenting patient-derived resources that include all patients with PDAC.

## Materials and Methods

### Patients and Ethics Statement

The study prospectively enrolled consecutive 35 patients with liver metastasis who visited the Pancreatobiliary Cancer Clinic at the National Cancer Center, Korea. All patients provided written informed consent. The study protocols were approved by the Institutional Review Board of the National Cancer Center of Korea (Approval number of IRB: NCC-15-054 and NCC-16-011).

All animal studies were reviewed and approved by the Institutional Animal Care and Use Committee (IACUC) of the National Cancer Center Research Institute (NCCRI) (NCC-16-247). The NCCRI is a facility accredited by the Association for Assessment and Accreditation of Laboratory Animal Care International (AAALAC International) and abides by the guidelines of the Institute of Laboratory Animal Resources (ILAR) (accredited unit—NCCRI: unit number: 1392).

### Tumor Specimens

All procedures used to obtain tumor specimens were performed by experienced radiologists. Needle biopsies were performed using a freehand technique under real-time ultrasound guidance (Acuson Sequoia, Siemens Healthcare; or Logiq E9, GE Healthcare). Usually, two biopsy samples were obtained from each liver mass using an 18-gauge biopsy device. One biopsy sample was submitted for pathologic examination. A pathologist was not physically present during biopsies. The number of samples obtained from each mass was based on operator preference and the appearance of the biopsy cores. Specimens were transferred to cold phosphate-buffered saline (PBS) containing 1% Zell Shield (Biochrom AG, Germany).

### Establishment of Patient-Derived Orthotopic Xenograft (PDOX) Models

Female *Hsd:athymic nude-Foxn1* mice aged 5–8 weeks (Harlan Laboratories, Inc., Indianapolis, IN, USA) were housed in a specific pathogen free (SPF) environment under controlled conditions of light and humidity and were allowed food and water *ad libitum*. Mice were anesthetized using 2% isoflurane in 100% oxygen. A small transverse incision (1 cm) was made on the left flank of each mouse, exposing the pancreas, and a 3- to 5-mm incision was made in the tail of pancreas. A pooled PLB specimen was inserted into this incision, which was sealed using 6-black silk surgical sutures (Ailee, Korea). The tail of the pancreas was subsequently returned to the abdominal cavity, and the incision closed with sutures.

Tumors attaining a volume of 1,000~1,500 mm^3^ were removed and divided into 3 × 3 × 3 mm^3^ cubic fragments. Fragments were used for systematic maintenance and cryopreservation, histopathological analysis, preparation of DNA or RNA for next generation sequencing (NGS) analysis, and culture of organoids. Tumors of PDOX models were periodically monitored using a superconducting magnet system (7T BioSpec 70/20USR, Bruker, Germany) with T1-, T2-, and T2^*^-weighted (T2^*^w) imaging techniques.

### Histopathological Analysis

Tumors were fixed in 4% paraformaldehyde for 24 h and embedded in paraffin. Both human and mouse tumor tissues were sectioned at 10 μm thickness and stained with hematoxylin and eosin (H&E). All samples were evaluated by a board-certified pathologist specializing in architecture and desmoplastic appearance. All images were captured by an Aperio digital pathology slide scanner (Aperio Technologies, Inc., USA) and histology analyzed using Image Scope software version 11.

### Comprehensive Cancer Panel

Genomic DNA of patients (F0) and PDOXs was extracted from blood samples using QIAamp blood DNA mini kits (Qiagen, Valencia, CA) and from formalin-fixed paraffin embedded (FFPE) PLB samples using QIAamp DNA FFPE tissue kits (Qiagen). To prevent sequencing artifacts, DNA samples were treated with uracil-DNA glycosylase (UDG) prior to amplification. Targeted panel sequencing was performed with the Ion AmpliSeq Comprehensive Cancer Panel covering 409 genes (Ion Torrent, Life Technologies, Carlsbad, CA). Libraries were prepared for sequencing according to the manufacturer's instructions, and the quality of the libraries was determined using a 2100 Bioanalyzer (Agilent Technologies, Santa Clara, USA). Sequencing was performed using the platform Nextseq 500 System platform, with 2 × 151 bp paired end sequencing runs (Illumina Inc., San Diego, CA).

### Alignment and Detection of Somatic Mutations

The QC sequence was determined using FastQC 0.11.5 and mapped to the combined reference genome, which included both the human (GRCh37) and mouse (mm10) genomes, using bwa 0.7.12. BAM files were realigned with the Genome Analysis Toolkit 3.5 (GATK) Indel Realigner, and base quality scores were recalibrated by the GATK base quality recalibration tool. The reads mapped to the human reference genome were extracted, whereas those mapped to the mouse genome were removed, using Samtools 1.3.1.

The matched analysis-ready normal and tumor BAM files were analyzed by Mutect 1.1.7 and Strelka 1.0.14 to detect appropriate somatic mutations. Functional information about the variants was annotated using Oncotator 1.8.0.

### Droplet Digital Polymerase Chain Reaction (ddPCR)

KRAS mutations in genomic DNA extracted from primary tumor samples (F0) and each generation of PDOX (F1 ~ F3) tissue samples were assessed by droplet digital PCR (ddPCR) (16) using a specific primer of KRAS G12V and G12D for PDOX models and using a KRAS screening multiplex droplet digital PCR Kit for organoid, which covers the mutations in codons 12 and 13 (i.e., G12A, G12C, G12D, G12R, G12S, G12V, and G13D) (Biorad-Laboratories, Hercules, CA, USA), and a QX200 Droplet Digital PCR System (Biorad-Laboratories). After amplifying to the endpoint (40 ~ 55 cycles) the ddPCR results were analyzed using QuantaSoft software (Biorad-Laboratories).

### Short Tandem Repeat

Short tandem repeat (STR) analysis was performed at 10 loci on different chromosomes to verify that the PDOX, PDOX-derived cells, organoid samples analyzed were derived from each patient. Target DNA (10 ng) was amplified by multiplex polymerase chain reaction (PCR) using fluorescent dye linked primers for STR loci (TH01, D21S11, D5S818, D13S317, D7S820, D16S539, CSF1PO, AMEL, vWA, TPOX). Amplification was performed using a GenePrint® 10 system Kit (Promega, WI, USA) according to the manufacturer's instructions. Samples were run on an ABI 3730 DNA Analyzer (Thermo Fisher Scientific) and analyzed in GeneMapper v4.0.

### Culture of PDOX-Derived Organoids

Cells were isolated from a viable portion of tumor tissue in PDOX. Single cell suspensions were obtained using a combination of mechanical dissociation and enzymatic degradation of the extracellular matrix (gentle MACS Dissociators, Milteny Biotec, Germany). PDOX-derived cells were cultured in RPIM-1640 (Hyclone) supplemented with 10% fetal bovine serum (FBS, Gibco) and Zell Shield. Contaminating stromal cells were removed by differential trypsinization or selective scraping of the plates, as necessary. These protocols were repeated until pure tumor cell populations were obtained.

For organoid culture, PDOX-derived cells at first passage were dissociated and mixed with 2 μl growth factor reduced (GFR) Matrigel (BD Bioscience, USA) containing 1 × 10^3^ cells per well of a 96-well plate. After the Matrigel hardened, the cells were incubated with advanced DMEM/F12 medium (Invitrogen, USA) containing B27 (Invitrogen, USA), N-acetylcysteine (Sigma, USA), EGF (PeproTech, USA), FGF-10 (PeproTech, USA), R-spondin 1, and Noggin (PeproTech, USA). Medium was changed every 3 days.

### Immunofluorescence Staining in Organoid

Organoids from PDOX of diameter 100 ~ 200 μm formed after 14 days in culture. To embed these organoids in paraffin blocks, the medium was removed and the cells were washed with PBS. Agarose was added to a concentration of 20%, followed by incubation for 20 min at room temperature. The hardened organoid-agarose gels were fixed in 4% paraformaldehyde for 30 min, followed by sectioning and incubation with the appropriate antibodies.

### Drug Response Test in Organoids

Gemcitabine-HCl was obtained from Dong-A ST Co., Ltd., Seoul, Korea and albumin-bound paclitaxel (Abraxane®) was obtained from Celgene Corporation, NJ, USA. PDOX-derived organoids (1 × 10^3^ cells/well) were seeded in 96-well plates as described. After 5 days in culture, the medium was replaced with fresh medium containing DMSO, gemcitabine, and albumin-bound paclitaxel (Abraxane), followed by incubation for 7 days. Cell growth was analyzed by microscopy and by measuring ATP concentrations with Cell Titer-Glo® 3D Viability assay kit (Promega Corporation, WI, USA).

The synergistic effects of combination treatment were assessed by calculating both the combination index (CI) and Bivariate Response to Additive Interacting Doses (BRAID) model. CI was calculated dose-response curve parameters using the CompuSyn program, as a CI <1 indicates synergism, a CI = 1 indicates an additive effect, and a CI > 1 indicates antagonism (17). In BRAID model, k values <0 indicated antagonism; k = 0 indicated additive activity; and k > 0 indicated synergy (18).

### *In vivo* Drug Response Test

For the set of drug response studies, mice bearing orthotopically pancreatic PDOX-2 tumors from patient 2 were staged to approximately 100 mm^3^ prior to initiation of treatments and randomized to four groups of five mice each. The experimental groups included the control group (PBS as vehicle, i.v.) gemcitabine (150 mg/kg of bodyweight, once every 3 days, i.v.), Abraxane (25 mg/kg of bodyweight, once every 3 days, i.p.), and the combination group (the first treatment of 150 mg/kg of gemcitabine, followed by 6 hours after the second treatment of 25 mg/kg of Abraxane, once every 3 days). Mice were monitored every 10 days by magnetic resonance imaging (MRI). The tumor volume was assessed in each MRI examination independently by region of interest (ROI)-based volumetry. For the ROI-based measurement, the entire tumor region identified and traced on the MRI workstation on all T2-weighted sagittal imaging slices throughout the tumor. A 3D ROI-based volume was calculated by the summation of all tumor areas in each slice and multiplication by the slice profile (0.8 mm thickness plus 0.3 mm gap); tumor volume = (ROI A1 + ROI A2 + ROI A3 … +ROI An) × 1.1 mm. After 75 days, mice were euthanized, and the tumors were excised, weighted, and prepared for paraffin embedding.

### Statistical Analysis

All statistical analyses were performed with GraphPad Prism 5 (GraphPad Software, Inc., CA). Differences in tumor volumes and growth rates among the mouse xenograft models were analyzed by *t*-test. A *P* value <0.5 was considered to indicate a statistically significant difference.

## Results

### Patient Characteristics and Establishment of Patient-Derived Orthotopic Xenografts (PDOX) From PLB Samples

The study prospectively enrolled consecutive 35 patients with liver metastasis who visited the Pancreatobiliary Cancer Clinic at the National Cancer Center, Korea. Of these 35 patients enrolled in the study, 22 (62.9%) were men and 13 (37.1%) were women, with a median age of 63.1 years (Table 1). Most patients (26/35; 74%) had poorly differentiated tumors and high CA19-9 concentrations (>100 U/ml). First-line chemotherapy in 15 (42.9%) patients consisted of gemcitabine-based combinations, including gemcitabine plus Abraxane, gemcitabine plus capecitabine, and gemcitabine plus erlotinib. Six (17.1%) patients were treated with FOLFIRINOX, whereas one received gemcitabine monotherapy. Median follow-up time was 4.1 months (range, 0.7–24.3 months) and median time-to-progression was 3.2 months (range, 0.2–14.1 months) (Supplementary Table 1, Supplementary Figure 1).

**Table 1:** Patient characteristics (*n* = 35).

**Characteristics**		**Total (*n*)**
**GENDER**		
	Male	22
	Female	13
**AGE**		
	Median (range)	64 (40–80)
**ECOG**		
	No	22
	Yes	13
**LOCATION**		
	Head	10
	Body and tail	25
**TUMOR SIZE (CM)**		
	Median (range)	3.7 (2.0–7.5)
**DIFFERENTIATION**		
	Moderately	9
	Poorly	26
**CA19-9 BASELINE**		
	Median (range)	2195 (5–30900)
	≤100	9
	>100	26
**CEA BASELINE**		
	Median (range)	9.4 (1.7–241.9)
	≤5	12
	>5	23
**TIME-TO-PROGRESSION**		
	Median (range)	192 (5–423)
**SURVIVAL**		
	Median (range)	123 (22–660)
**FOLLOW-UP DURATION**		
	Median (range)	96 (5–660)

**Figure 1:**
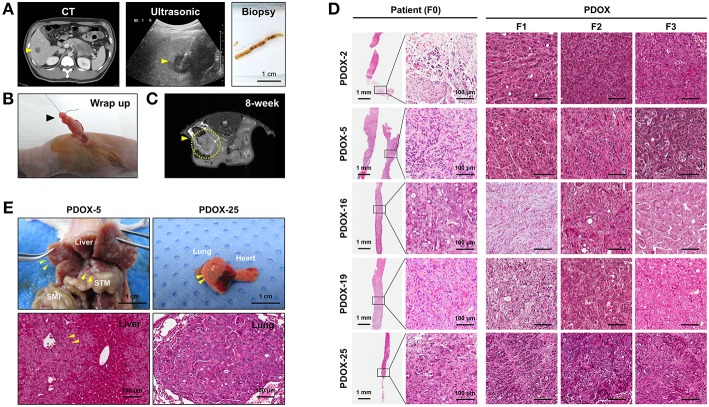
Establishment of PDOX from a percutaneous liver biopsy (PLB) of metastatic pancreatic cancer. **(A)** Preparation of needle biopsy. Abdominal CT, ultrasonography and image of a patient who underwent PLB. Scale bar = 1 cm. **(B)** Method for orthotopic implantation of a needle biopsy sample. An incision and wrapping were made in the tail of an exposed pancreas, followed by suturing of the biopsy. **(C)** Monitoring of tumor volume by magnetic resonance imaging (MRI) in PDOX models. **(D)** Retention of histopathological features of primary tumors by PDOX tumors. H&E staining showed similar histological morphologies of PDOX tumors (F1, F2, and F3) and the PLB sample from a patient (F0). Scale bars = 1 mm, 100 μm. **(E)** Top, Distant metastasis in the PDOX model. Images from laparotomy of the PDOX 3 months after orthotopic implantation of a PLB sample. The yellow arrows indicate liver metastases. Scale bar = 1 cm. SMI, small intestine; STM, stomach. Scale bar = 1 cm. Bottom, H&E staining of the metastatic liver (left bottom) and lung (right bottom) in the F1 xenograft were similar to that of the original primary tumor shown in **(D)**. Scale bar = 100 μm.

Tumor samples used in this study were derived from ultrasound-guided biopsies of liver metastases in patients with PDAC (Figure 1A). Biopsy specimen for transplantation is stored in cold buffer on ice immediately after obtaining it and implanted to mouse within 30 min for reducing hypoxia and preservation of viability. PDOX models were established by implanting PLB samples orthotopically into the pancreases of athymic nude mice using an incision and wrapping method (Figure 1B), and the growth of these tumors was monitored by MRI every 2 weeks (Figure 1C). Of the 35 tumor specimens, 15 (43%) engrafted successfully, reaching a size of 1,000 ~ 1,500 mm^3^ within a median 107 days (range, 52–348 days). Median time to engraftment tended to be shorter for poorly than for moderately differentiated tumors (91.5 vs. 329 days, *P* = 0.0513) (Table 2).

**Table 2:** Clinicopathologic characteristics and time-to-engraftment.

**Characteristics**			**Number (%)**	**Time to PDX (days)**	***p*-value**
				**Median (range)**	
**GENDER**					
	1:00	Male	11 (73.33%)	94 (52–329)	0.1332
	2:00	Female	4 (26.67%)	237 (89–348)	
**ECOG**					
	0:00	No	9 (60.00%)	94 (63–329)	0.7683
	1:00	Yes	6 (40.00%)	154 (52–348)	
**LOCATION**					
	1:00	Head	6 (40.00%)	158 (80–329)	0.5959
	2:00	Body and tail	9 (60.00%)	107 (52–348)	
**DIFFERENTIATION**					
	2:00	Moderate	3 (20.00%)	329 (126–348)	0.0513
	3:00	Poor	12 (80.00%)	91.5 (52–307)	
**CA19-9 BASELINE**					
	0:00	≤100	5 (33.33%)	126 (80–329)	0.7595
	1:00	>100	10 (66.67%)	100.5 (52–348)	
**CEA BASELINE**					
	0:00	≤5	5 (33.33%)	85 (52–222)	0.0982
	1:00	>5	10 (66.67%)	146.5 (63–348)	

Histologically, PDOXs (F1, F2, and F3) closely resembled their parent tumors (F0) (Figure 1D and Supplementary Figure 2A). Two PDOX models of tumors with aggressive biology spontaneously metastasized to different organs. Multiple liver (PDOX-5) and lung (PDOX-25) metastases were observed as well as original implant-site pancreatic tumors (Figure 1E). In addition, a metastatic nodule was observed in the diaphragm and hydrops abdominis was present in the abdominal cavity (PDOX-3) (Supplementary Figure 2B). Serial *in vivo* passage increased the growth rates of PDOX tumors, with median times to tumorigenicity of F1, F2, and F3 PDOXs being 168.2, 72, and 62.2 days, respectively (Supplementary Figure 3).

### Molecular Characterization of Tumors and PDOX Models

Genetic alterations in DNA purified from FFPE of both primary tumors and PDOX models were analyzed by targeted sequencing using a comprehensive cancer panel (CCP), which included 409 cancer-associated genes, including both tumor suppressor genes and oncogenes. When the tumor cellularity of patient samples was insufficient, the passaged xenografts (F1, F2, and F3) were molecularly characterized. In general, PDOXs showed high genomic stability, at least through the first three passages.

**Figure 2:**
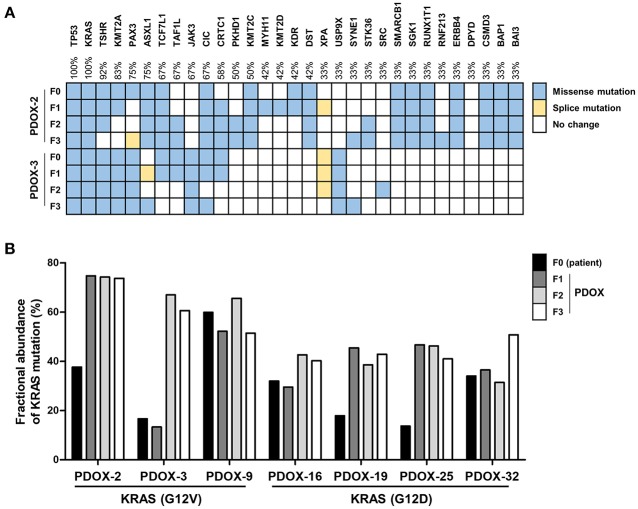
Retention of genetic alterations between primary and PDOX tumors. **(A)** Heat maps depicting the overall mutations of a paired set of genes in PDOX-2 and PDOX-3 from CCP data. **(B)** Comparison of *KRAS* mutation fractional abundances from ddPCR that was used with specific primers for G12V or G12D mutation detection, between primary and passaged PDOXs tumors.

Ninety two and fifty seven mutations in primary tumors of PDOX-2 and PDOX-3, respectively, were also detected in their corresponding F1 PDOXs (PDOX-2, 85 mutations; PDOX-3, 58 mutations), suggesting that PDOXs were able to recapitulate the majority of mutations in primary tumors. Full concordance in representative somatic mutations, including in TP53 and KRAS, was observed between primary tumors (F0) and PDOX (F1, F2, and F3) (Figure 2A). KRAS, one of the most frequently mutated oncogenes, observed in 90% of PDACs, was found to be mutated at codon 12 in these samples, with the most common mutations being G12D and G12V, with frequencies of 51 and 30%, respectively. Moreover, this method, along with digital PCR, was utilized to detect molecular abnormalities in PDOX models and their variations during xenograft passaging (Figure 2B). Droplet digital PCR showed that the KRAS mutations present in primary tumors were retained by their corresponding PDOXs, with higher fractional abundance (Figure 2B).

### Generation and Characterization of PDOX-Derived Organoids

Successful generation of tumor organoids from tumor tissue requires a large population of cancer cells and exclusion of normal cells (Supplementary Figure 4). Organoids were generated using the first passage of epithelial cancer cells isolated from F1 PDOX tumor tissue. Use of these cells may enhance cancer cell selection by removing fibroblast and normal cells without loss of heterogeneity.

**Figure 3:**
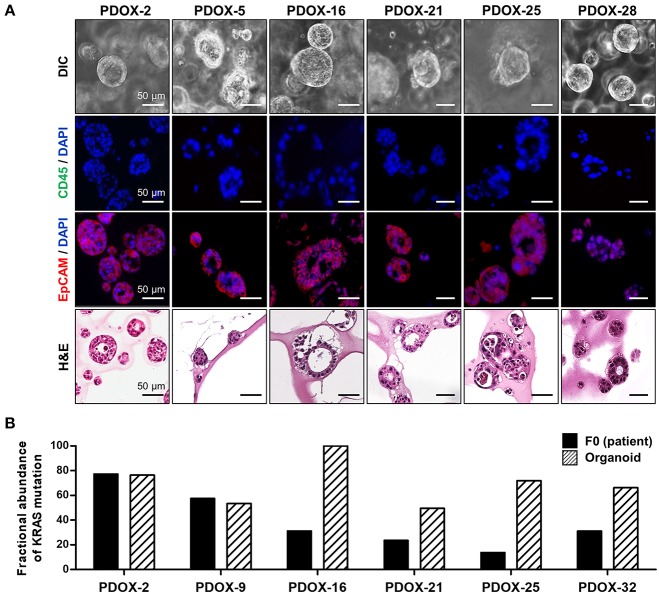
Characterization of PDOX-derived organoids. **(A)** Representative organoid images using DIC, EpCAM staining using immunofluorescence and H&E. **(B)** Expression of mutated KRAS in PDOX and derived organoids. Fractional abundance of KRAS mutations by ddPCR in primary tumors and PDOX-derived organoids. Multiplex *KRAS* mutation detection kit that encompasses 7 common mutations (G12A, G12C, G12D, G12R, G12S, G12V, and G13D) was used in this experiment.

Organoid models were successfully formed from mixtures of cancer cells and a tiny droplet of Matrigel grown in pancreatic cancer-specific organoid medium for 6–10 days. Organoids showed rearrangement of cells and had a ductal morphology similar to human pancreatic cancer (Figure 3A). Organoid models varied in shape and size, suggesting that they mimic the morphologic heterogeneity of the primary tumors. Organoids were shown to originate from epithelial cancer cells by staining with antibodies to EpCAM, a positive cancer cell marker, and CD45, a macrophage marker not expressed by cancer cells (Figure 3A). From droplet digital PCR, the fractional abundance of KRAS mutations in almost organoids, except PDOX2 and 9, was higher than in corresponding primary patient tumors, indicating driver-gene alteration such as KRAS mutation was retained and enriched during organoid generation and passages (Figure 3B). In addition, short tandem repeat (STR) profiling with 10 loci demonstrated that all PDOX-derived models were unique and matched to the original patient, as shown in DNA fingerprinting results (Table 3).

**Table 3:** Short Tandem Repeat (STR) profiling of PDOX and organoid.

	**Locus**	***TH01***	***D21S11***	***D5S818***	***D13S317***	***D7S820***	***D16S539***	***CSF1PO***	***AMEL***	***vWA***	***TPOX***
PDOX-2	F0	9	30	10, 12	11, 12	7, 9	10	12, 13	X, Y	18, 19	8, 11
	F1	9	30	10, 12	11, 12	7, 9	10	12, 13	X	18, 19	8
	F2	9	30	10, 12	11, 12	7, 9	10	12, 13	X	18, 19	8
	F3	9	30	10, 12	11, 12	7, 9	10	12, 13	X	18, 19	8
	Cell	9	30	10, 12	11, 12	7, 9	10	12, 13	X	18, 19	8
	Org.	9	30	10, 12	11, 12	7, 9	10	12, 13	X	18, 19	8
PDOX-25	F0	7	30, 31.2	12, 13	10, 12	10, 11	9, 10	11, 12	X, Y	15, 17	8
	F1	7	30, 31.2	12, 13	10, 12	10, 11	9, 10	11, 12	X, Y	15, 17	8
	F2	7	30, 31.2	12, 13	10, 12	10, 11	9, 10	11, 12	X, Y	15, 17	8
	F3	7	30, 31.2	12, 13	10, 12	10, 11	9, 10	11, 12	X, Y	15, 17	8
	Cell	7	30, 31.2	12, 13	10, 12	10, 11	9, 10	11, 12	X, Y	15, 17	8
	Org.	7	30, 31.2	12, 13	10, 12	10, 11	9, 10	11, 12	X, Y	15, 17	8
PDOX-28	F0	9	30, 32.2	10, 12	11, 12	8, 11	10, 12	11, 12	X, Y	16, 17	8
	F1	9	30, 32.2	10, 12	12	8, 11	10, 12	11, 12	X, Y	16, 17	8
	F2	9	30, 32.2	10, 12	12	8, 11	10, 12	11, 12	X, Y	16, 17	8
	F3	9	30, 32.2	10, 12	12	8, 11	10, 12	11, 12	X, Y	16, 17	8
	Cell	9	30, 32.2	10, 12	12	8, 11	10, 12	11, 12	X, Y	16, 17	8
	Org.	9	30, 32.2	10, 12	12	8, 11	10, 12	11, 12	X, Y	16, 17	8
PDOX-32	F0	6, 7	28, 29	11	10, 13	11	9, 11	10, 12	X	14, 17	11
	F1	6, 7	28	11	10, 13	11	9, 11	10	X	14, 17	11
	F2	6, 7	28	11	10, 13	11	9, 11	10, 12	X	14, 17	11
	F3	6, 7	28	11	10, 13	11	9, 11	10, 12	X	14, 17	11
	Cell	6, 7	28	11	10, 13	11	9, 11	10, 12	X	14, 17	11
	Org.	6, 7	28	11	10, 13	11	9, 11	10, 12	X	14, 17	11

**Figure 4:**
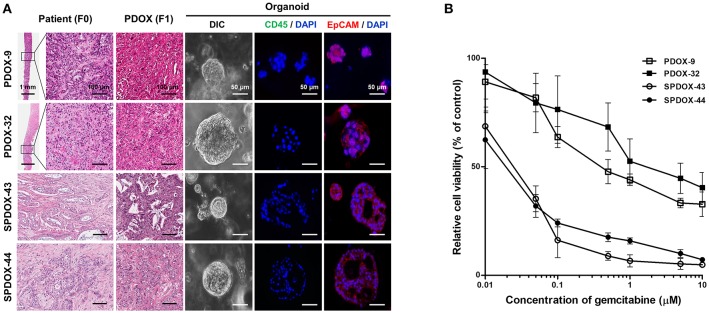
Correlation of drug sensitivity between PDOX-derived organoids and corresponding patients. **(A)** Representative images of organoids derived from PDOX-9 and PDOX-32 which were generated from gemcitabine-resistant metastatic patients, and SPDOX-43 and SPDOX-44 from gemcitabine-sensitive resectable patients. Scale bar in H&E = 100 μm, Scale bar in DIC and immunofluorescence = 50 μm. **(B)** Response curves of organoids to gemcitabine. Organoids were treated with gemcitabine for 7 days, followed by measuring ATP concentrations for cell viability assay as described in “Materials and Methods”.

To determine whether PDOX-organoids could predict responsiveness or resistance to drugs of primary tumors, we randomly selected two organoids from SPDOX-43 and−44, which were derived from surgical primary tumor of gemcitabine-sensitive patients, respectively, and two organoids from PDOX-9 and−32, which were derived from gemcitabine-resistant metastatic tumors (Figure 4A). As expected, the sensitivity of the organoids to gemcitabine correlated with the sensitivity of the corresponding tumors. SPDOX-43 and SPDOX-44 organoids were very sensitive to gemcitabine, even at very low doses of 0.01 μM (Figure 4B). In contrast, PDOX-9 and−32 organoids were highly resistant to gemcitabine. These findings indicate that this PDOX-derived organoid system may be a reliable predictor of responses of advanced pancreatic tumors to treatment.

### PDOX-Organoids' Ability to Predict Tumor Response to Combination Chemotherapy

**Figure 5:**
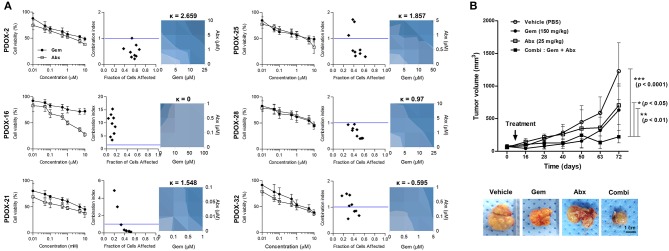
Response of PDOX and PDOX-derived organoids to gemcitabine and Abraxane. **(A)** Response curves of patient-derived organoids to gemcitabine (left) and the combination of gemcitabine and Abraxane. The synergic effect of the combination of these two agents was assessed by comparing the effects of the combination of the two drugs with each alone using the CompuSyn program, with results reported as the combination index (CI) as a function of the fraction affected (Fa). Drug synergy was also calculated using BRAID models, with k > 0 indicating synergy, k = 0 indicating an additive effect, and k < 0 indicating drug antagonism (right-blue). Error bars indicate standard deviations (SD) and all experiments were performed in triplicate. **(B)** *In vivo* drug efficacy testing of combination therapy using PDOX models. Combination effects of gemcitabine and Abraxane were tested in PDOX-2 models. Average tumor volumes of treated groups are plotted in graph and representative tumors after treatment are shown (bottom). Vehicle; PBS, Gem; gemcitabine, Abx, Abraxane; Combi, gemcitabine plus Abraxane. ***p < 0.0001, **p < 0.01, *p <0.05. Scale bar = 1 cm.

We also tested the effect on six organoids of treatment with a combination of gemcitabine and Abraxane, as well as with each individual agent (Figure 5A). Almost organoids were resistant to gemcitabine, with an IC50 >3 μM. The synergistic effect of combination treatment by measuring combination indexes (CI), with CI <1 indicating synergy and CI ≥1 indicating a lack of synergy. PDOX-2,−25, and−28 organoids with CI <1 were expected synergistic effect, in contrast to PDOX-16 and PDOX-32 organoids with CI > 1. Synergy was further assessed using a BRAID model to be clear the response parameter of two-drug combination, in which k values <0 indicated antagonism; k = 0 indicated additive activity; and k > 0 indicated synergy. Gemcitabine and Abraxane had synergistic effects against PDOX-2,−21,−25, and−28 organoids, with k values of 2.559, 1.548, 1.857, and 0.97, respectively. In contrast, these two agents had antagonistic effects against PDOX-32 organoids, with a kappa value of −0.595, a response corresponding to that of the primary tumor, which was resistant to the combination of gemcitabine and Abraxane. PDOX-2-derived organoid showed obviously the synergistic effect of combination treatment from both CI index and BRAID model. Therefore, for *in vivo* validation, the PDOX-2 PDX model was treated with gemcitabine (150 mg/kg), and/or Abraxane (25 mg/kg) (Figure 5B). The average tumor volumes in the control group, gemcitabine group and Abraxane group were 1228.70 ± 435.20 mm^3^, 633.10 ± 400.90 mm^3^, and 705.00 ± 295.00 mm^3^ at day 72, while those in combination group reached only 222.52 ± 93.95 mm^3^. Combination treatment showed the very significant synergy effect (vehicle vs. combination treatment, *p* < 0.0001), in contrast, the p values between the vehicle and the gemcitabine groups, and between the vehicle and the Abraxane groups were not significant. Further, combination treatment inhibited tumor growth significantly compare with gemcitabine or Abraxane group, respectively (*p* < 0.01, *p* < 0.05) (Figure 5B). These findings support the *in vitro* and *in vivo* ability of our PDOX-organoid paired platform to predict primary tumor responses to combination chemotherapy.

## Discussion

Patient-derived models are replacing immortalized cancer cell lines in preclinical studies of anti-cancer drugs (19). PDX and organoid models better reflect primary tumors because of the comprehensive heterogeneity of their individual cancer cells (7). To our best knowledge, the present study is the first to test a PDOX-derived organoid model system for metastatic PDAC, a type of tumor specimen with low cellularity. In general, generation of pancreatic cancer PDX by subcutaneous implantation has been limited to the use of surgically resected material as the source of viable tumor cells. These samples are not representative of all PDACs, because approximately 85% of pancreatic cancer patients are ineligible for surgical resection of their tumors (8, 9, 20). This study describes the establishment of an effective PDOX model of PDAC using PLB, with the majority of PDAC patient-derived samples were engrafted successfully. They also retained the genetic alterations and histopathological features of their primary tumors. Orthotopic implantation into the pancreas can enhance the success rate and provide a proper microenvironment for patient-like tumor behavior. Moreover, we have observed metastatic spread of some PDOXs to the liver and other intra-abdominal organs (Figure 1E, Supplementary Figure 2B). Second generation (F2) xenograft models were made using tissue samples from the tumor on pancreas and metastatic tumor on liver in F1 PDOX These second generation PDOXs retained the histopathological features of their primary tumors, including poor differentiation. Although the time-to-engraftment was faster for the second-generation tumor (1.5 months) than for the primary tumor (3 months), none of the second passage PDOX models metastasized (data not shown). Despite its rapid growth at the primary site, the time allowed may have been insufficient for metastasis of the latter.

PDX models has some hurdle to be an usual preclinical assay system for large-scale drug discovery despite of many advantages, as they are not suitable for high-throughput screens, as well as involving costly and lengthy processes to sufficient animal numbers for drug treatment, although PDX-guided treatment showed an impressive 88% response rate in a small sample size study (21). However, PDX models can confirm findings from high-throughput *in vitro* studies. To overcome this problem and produce the reliable data using patient-derived resources, we introduced PDOX-derived organoids.

Organoids can be derived directly from patients with various cancer types (11, 13, 22, 23), which required many trials to optimize media, including unique scaffolds and stromal components. In this study, we generated organoids using first passage-cancer cells isolated from F1 PDOX tissue, not from PDOX F1 tissue directly. This method, which enriched the population of cancer cells, enhanced the success rate of organoid formation, avoiding the formation of organoids from normal cells and excluding non-tumor cell populations. To minimize the loss of heterogeneity of adherent cell cultures, we used very early passage cancer cells, removing fibroblast populations. Isolation of pancreatic cancer cells from PDX models has been reported to allow expansion and preserve heterogeneity (24, 25). Adherent cells isolated from PDOX varied in shape and size and strongly expressed EpCAM, as well as showing greater tumor growth potential than the commercially available pancreatic cancer cell line CFPAC-1 (Supplementary Figure 5). Moreover, Short tandem repeat (STR) conducted for validation of the PDOX, PDOX-derived adherent cells, and organoid indicated that the original patient tumor and each model had identical characteristics (Table 3). Adherent cells isolated from PDOX may provide another resource for organoid generation and constitute a living biobank for cancer drug discovery.

The PDOX and subsequent organoid model system could predict tumor response to combination chemotherapy, the mainstay for treatment of patients with metastatic pancreatic cancer. In addition, the synergy of combination treatment with gemcitabine and albumin-bound paclitaxel in PDOX-2-derived organoids was observed in the PDOX-2 *in vivo* model, comparable to results evaluating the similar drug responses of parallel organoid cultures and PDX models (26). This parallel validation suggested the reliability of our platform. It may be difficult to implement precision medicine directly using this PDX-organoid platform in pancreatic cancer, considering the survival period and limitations of treatment drugs in metastatic inoperable pancreatic cancer patients. The value of our PDOX and organoid models established in this study are pre-clinical models that can reflect the clinical characteristics of patients at *in vitro* and *in vivo* levels; the contribution of these models is by acting as a library to investigate effective treatment strategies for pancreatic cancer based on the characteristics of numerous metastatic pancreatic patients.

**Figure 6:**
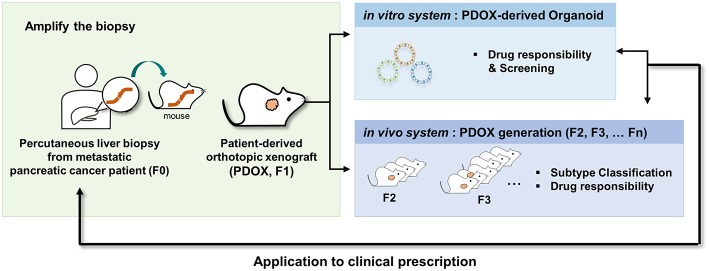
Schematic description of the overall platform for the establishment and utilization of pancreatic cancer PDOX and PDOX-derived organoids. Step 1, establishment of PDOX from a percutaneous liver biopsy of a patient with metastatic pancreatic cancer; step 2A, expansion and dissection of the molecular features of the PDOXs; step 2B, screening of candidate anticancer drugs using organoids from PDOXs; step 3, selective drug response of PDOX models; step 4, clinical application.

In conclusion, our results provide definitive evidence that PDOX and the subsequent organoid model system are applicable to metastatic pancreatic cancer, a disease with a dismal prognosis. These PDOX and organoid models mimicked the responses of primary tumors to drug treatment, suggesting that these models may be useful for translational research and drug discovery in PDAC (Figure 6).

## Data Availability

The raw data supporting the conclusions of this manuscript will be made available by the authors, without undue reservation, to any qualified researcher.

## Ethics Statement

The study prospectively enrolled consecutive 35 patients with liver metastasis who visited the Pancreatobiliary Cancer Clinic at the National Cancer Center, Korea. All patients provided written informed consent. The study protocols were approved by the Institutional Review Board of the National Cancer Center of Korea (Approval number of IRB: NCC-15-054 and NCC-16-011). All animal studies were reviewed and approved by the Institutional Animal Care and Use Committee (IACUC) of the National Cancer Center Research Institute (NCCRI) (NCC-16-247). The NCCRI is a facility accredited by the Association for Assessment and Accreditation of Laboratory Animal Care International (AAALAC International) and abides by the guidelines of the Institute of Laboratory Animal Resources (ILAR) (accredited unit—NCCRI: unit number: 1392).

## Author Contributions

S-YK, S-JP, SW, and Y-HKi: conceptualization. SC, A-RJ, MK, Y-SL, JI, J-WK, S-SH, Y-HKo, JL, WL, S-JP, EH, SW, and Y-HKi: data curation. SC, A-RJ, MK, SW, and Y-HKi: formal analysis. SC, A-RJ, MK, Y-SL, JI, J-WK, S-SH, Y-HKo, JL, WL, S-JP, EH, SW, and Y-HKi: investigation. SC, A-RJ, MK, S-YK, K-AY, S-JP, SW, and Y-HKo: validation. SC, SW, and Y-HKi: writing—original draft. SC, SW, and Y-HKi: writing—review and editing.

### Conflict of Interest Statement

The authors declare that the research was conducted in the absence of any commercial or financial relationships that could be construed as a potential conflict of interest.
