# Supplementary material for: Development of Patient-Derived Preclinical Platform for Metastatic Pancreatic Cancer: PDOX and a Subsequent Organoid Model System Using Percutaneous Biopsy Samples
Source: Front Oncol. 2019 Sep 13;9:875. doi: 10.3389/fonc.2019.00875 (PMC6753223; doi:10.3389/fonc.2019.00875)
Supplement: Supplementary file 1 [file Data_Sheet_1.docx]

Supplementary Material

# Supplementary Materials and Methods

## Cell culture

The pancreatic cancer cell line CFPAC-1, obtained from the American Type Culture Collection (ATCC, Rockville, MD, USA), was cultured in Iscove's Modified Dulbecco's Medium (IMDM, Gibco) supplemented with 10% fetal bovine serum (FBS) and 1% antibiotic-antimycotic solution (Gibco). Cells were maintained in a humidified incubator at 37°C and 5% CO2.

## Immunocytochemistry and flow cytometry analysis

PDOX-derived cells were cultured on poly-L-lysine coated eight-well chambered slides and fixed in 4% paraformaldehyde for 15 minutes at room temperature. The cells were incubated with 3% bovine serum albumin (BSA) for 30 minutes, washed, and incubated overnight at 4℃ with fluorescence conjugated primary antibodies against human-specific EpCAM (BD Bioscience, #347198, USA) and CD45 (BD Bioscience, #552848, USA) in 1% BSA. The slides were counterstained with Hoest33342 (Invitrogen, USA). Images were collected with a Zeiss Axioobserver. Z1 microscope and processed with ZEN software blue edition (Carl Zeiss, Germany).

Cells were dissociated from 6-well culture plates, stained with the same antibodies as above for 1 hr at 4℃, and assessed by flow cytometry. A total of 10,000 events per sample were acquired with FACS Verse (BD Bioscience), and data were analyzed with FlowJo data analysis software version 10 (FlowJo LLC, Ashland, OR).

## In vivo tumorigenicity of PDOX-derived cells

To estimate tumorigenic characteristics, the pancreases of mice were orthotopically injected with PDOX-derived cells (1x106 or 5x105 cells/50 µl) or CFPAC-1 cells (5x106 cells/50 µl) as control. Mice were inspected twice a week and tumor size was measured by MRI once a week. Mice were euthanized 6 weeks after the injection.

# Supplementary Figures


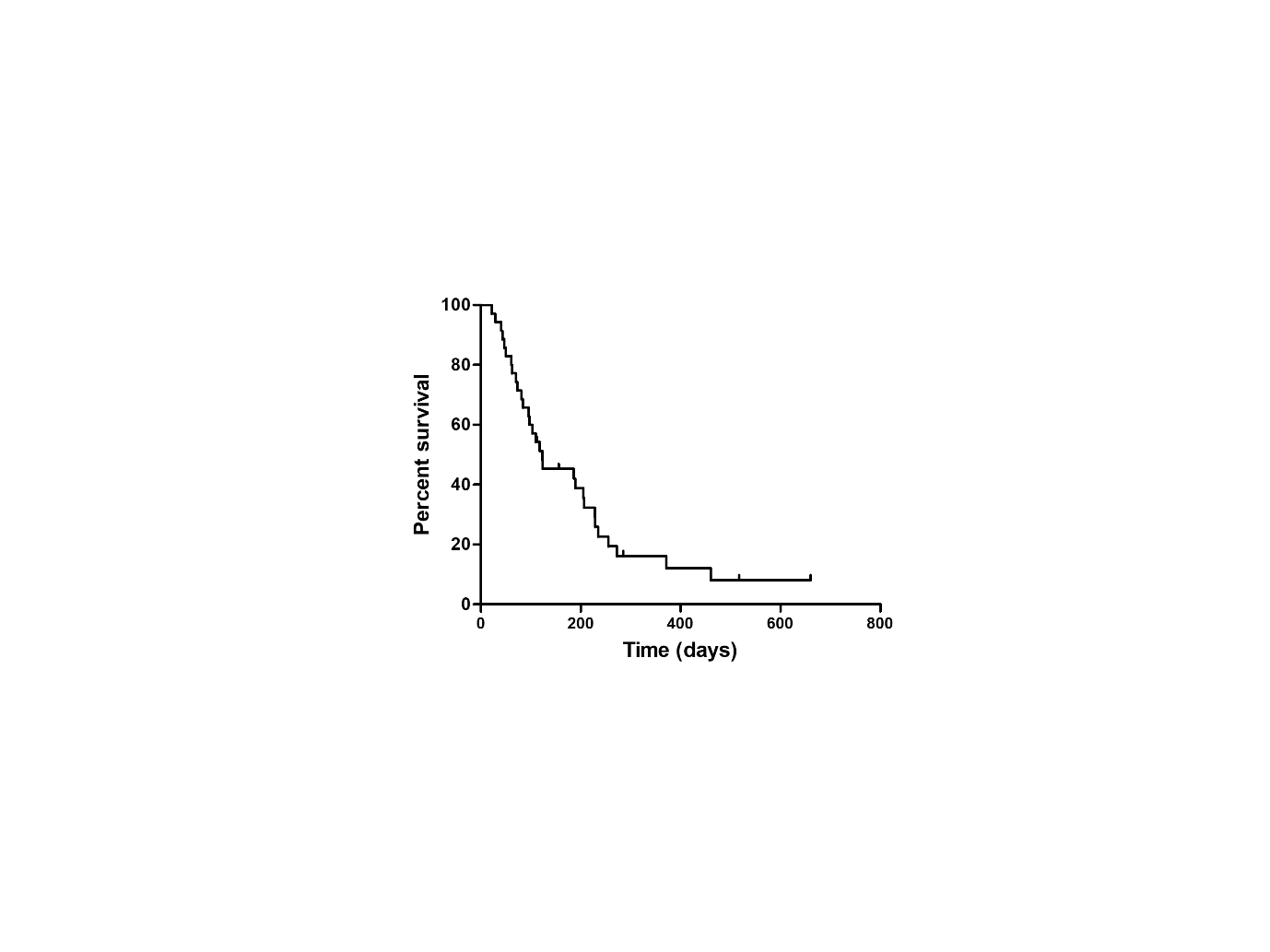


**Supplementary Figure 1.** Representation of the overall survival curve of patients with pancreatic ductal adenocarcinoma (PDAC), estimated by the Kaplan-Meier method.


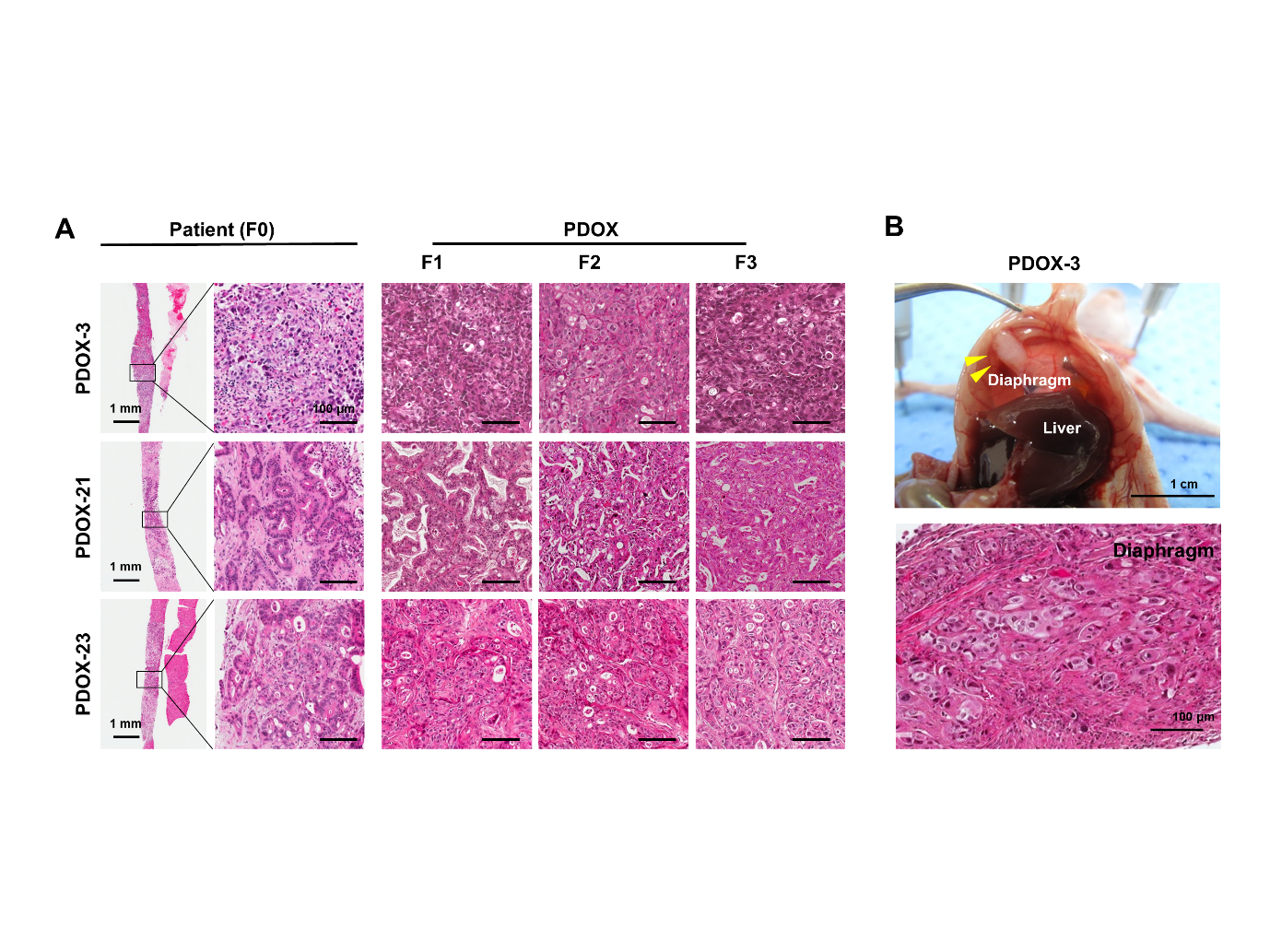


**Supplementary Figure 2.** Establishment of a patient-derived orthotopic xenograft (PDOX) from a percutaneous liver biopsy (PLB) obtained from a patient with metastatic pancreatic cancer. (A) H&E staining showing that the PDOX tumors (F1, F2 and F3) had retained the histopathological features of the original tumor (F0). Scale bar = 1 mm, 100 μm. (B) Distant metastasis in the PDOX model. Images from laparotomy of the PDOX-3, 3 months after orthotopic implantation of a PLB sample.


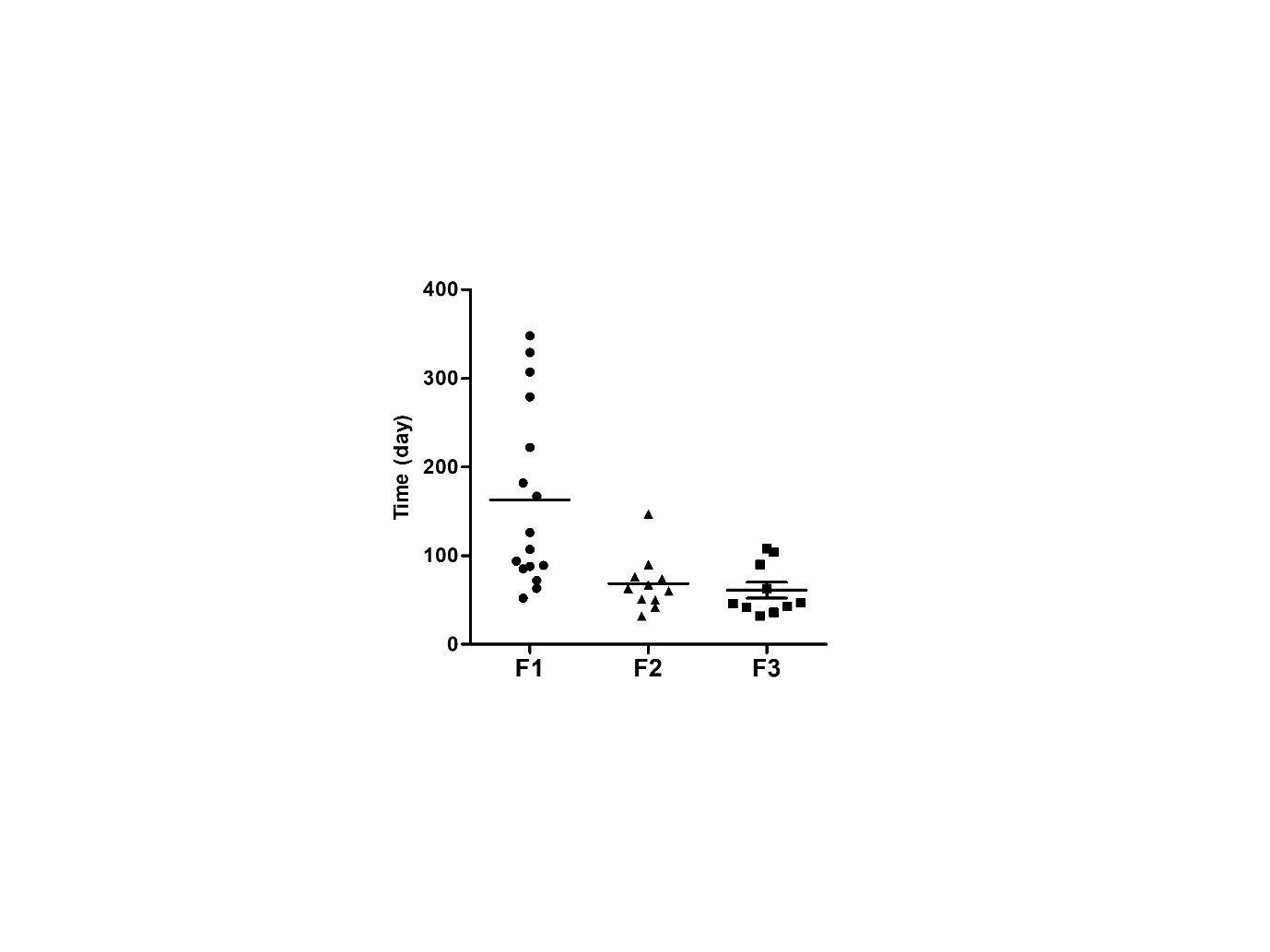


**Supplementary Figure 3.** Growth of successive passaged PDOX tumors, showing the times between tumor implantation and sacrifice (when the tumor volume reached 1000~1500 mm3) for all serial passages. Sixteen tumors were successfully passaged to the first generation (F1).


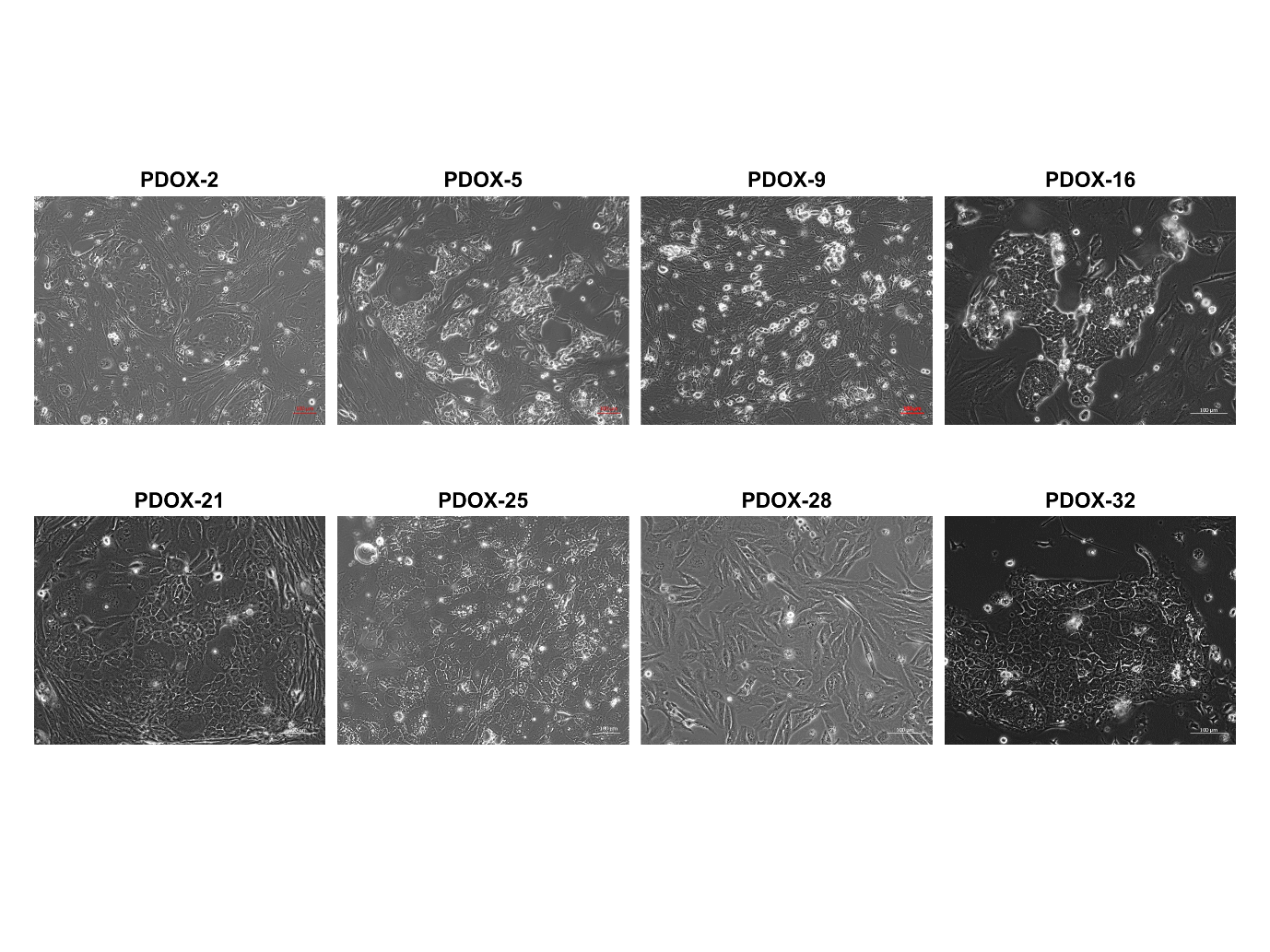


**Supplementary Figure 4.** Representative phase contrast images of first passaged-adherent cells isolated from each PDOXs F1. These were used for organoid generation as next step. Scale bar = 100 μm


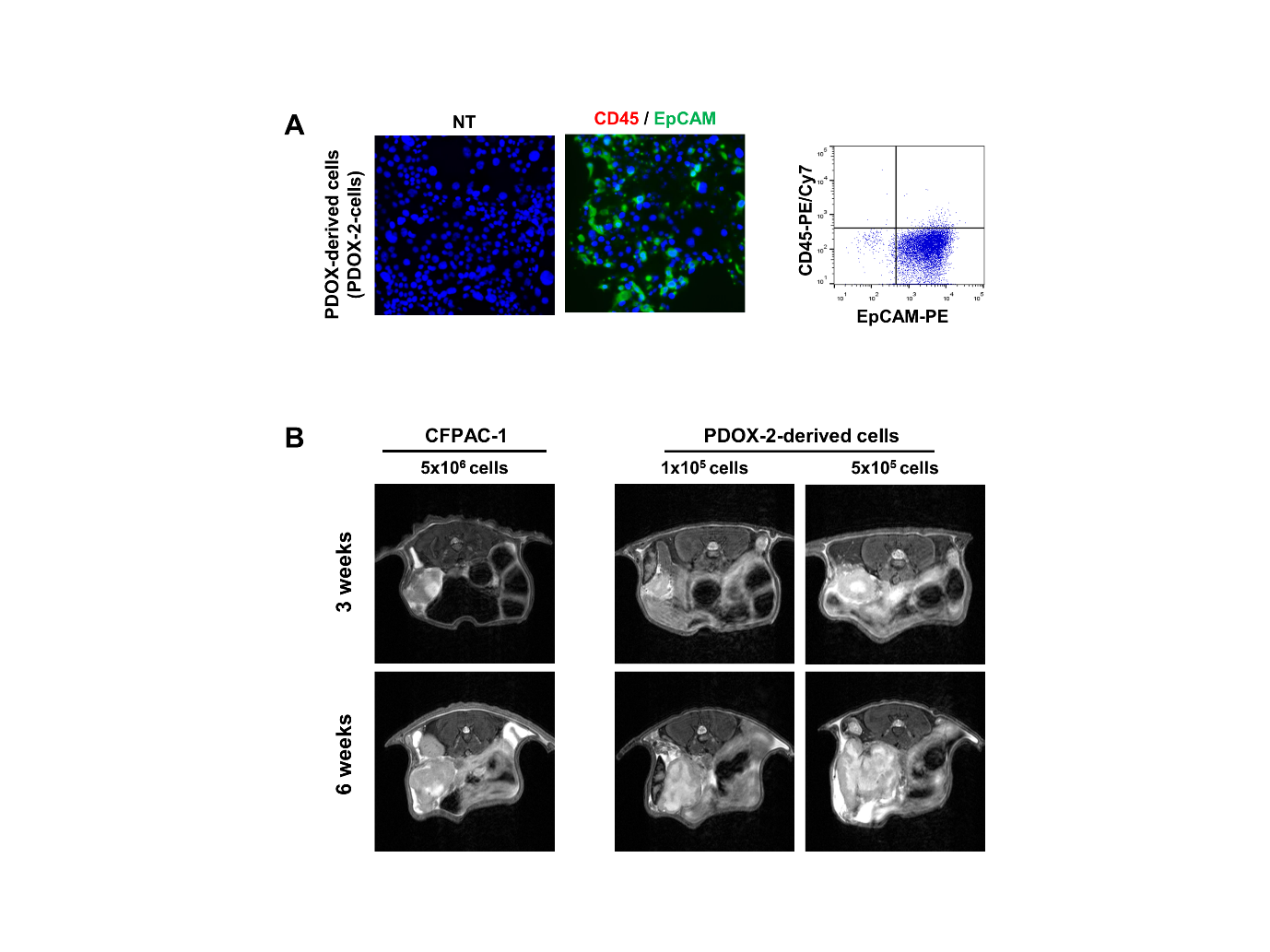


**Supplementary Figure 5.** Tumorigenic characteristics of PDOX-2-derived cancer cells. PDOX-2-derived cells were assessed by immunocytochemistry with antibodies to EpCAM and CD45 (A) and flow cytometry (B). (C) Tumorigenicity of PDOX-derived cells in orthotopic xenografts. the pancreases of mice were orthotopically injected with PDOX-derived cells (1x106 or 5x105 cells/50 µl) or CFPAC-1 cells (5x106 cells/50 µl) as control. Mice were inspected twice a week and tumor size was measured by MRI once a week

# Supplementary Table

## Supplementary Table 1. Characteristics of 35 patients and patient-derived orthotopic xenograft models

| Model Name | Gender | Age | Diagnosis | Location | Primary tumor size (cm) | Differentiation | Treatment | Death | Progression | Survival (days) | TTP (days) | PDOX (F1) |
| --- | --- | --- | --- | --- | --- | --- | --- | --- | --- | --- | --- | --- |
| PDOX-1 | M | 73 | PDAC | body and tail | 2.7 | PD | - | Y | - | 123 | 123 | Failure |
| PDOX-2 | M | 44 | PDAC | head | 4.2 | PD | FOLFIRINOX | Y | - | 124 | 63 | Success |
| PDOX-3 | M | 66 | PDAC | head | 2.3 | PD | Curative resection | Y | Y | 189 | 189 | Success |
| PDOX-4 | M | 40 | PDAC | body and tail | 6.5 | PD | FOLFIRINOX | Y | Y | 205 | 205 | Failure |
| PDOX-5 | F | 71 | PDAC | body and tail | 2.1 | PD | - | Y | - | 43 | 43 | Success |
| PDOX-6 | M | 66 | PDAC | body and tail | 3.4 | PD | Gemcitabine plus Capecitabine, GV1001 | Y | - | 97 | 94 | Failure |
| PDOX-7 | F | 75 | PDAC | body and tail | 4.2 | MD | Gemcitabine | Y | Y | 73 | 73 | Success |
| PDOX-8 | M | 63 | PDAC | head | 3.0 | MD | Gemcitabine plus Capecitabine | - | Y | 660 | 660 | Success |
| PDOX-9 | M | 49 | PDAC | body and tail | 3.7 | PD | Gemcitabine | Y | - | 63 | 63 | Success |
| PDOX-10 | F | 65 | PDAC | head | 2.0 | PD | Proton beam therapy | Y | Y | 255 | 225 | Success |
| PDOX-11 | F | 58 | PDAC | body and tail | 2.8 | MD | FOLFIRINOX | Y | Y | 228 | 217 | Failure |
| PDOX-12 | F | 49 | PDAC | body and tail | 6.8 | PD | FOLFIRINOX | Y | Y | 207 | 207 | Failure |
| PDOX-13 | M | 74 | PDAC | body and tail | 3.5 | PD | - | Y | - | 50 | 50 | Failure |
| PDOX-14 | F | 78 | PDAC | body and tail | 5.6 | MD | - | Y | - | 110 | 110 | Failure |
| PDOX-15 | F | 78 | PDAC | body and tail | 2.8 | PD | - | Y | - | 81 | 81 | Failure |
| PDOX-16 | M | 62 | PDAC | head | 7.5 | PD | - | Y | - | 229 | 229 | Success |
| PDOX-17 | M | 62 | PDAC | head | 3.6 | MD | Gemcitabine | Y | Y | 371 | 275 | Failure |
| PDOX-18 | F | 80 | PDAC | body and tail | 3.7 | PD | - | Y | - | 47 | 47 | Failure |
| PDOX-19 | M | 71 | PDAC | body and tail | 5.4 | PD | - | Y | - | 186 | 186 | Success |
| PDOX-20 | F | 70 | PDAC | head | 2.4 | PD | Gemcitabine plus Abraxane | - | Y | 517 | 510 | Failure |
| PDOX-21 | M | 63 | PDAC | body and tail | 6.0 | PD | - | Y | - | 70 | 70 | Success |
| PDOX-22 | M | 63 | PDAC | body and tail | 5.0 | PD | FOLFIRINOX | Y | Y | 460 | 405 | Failure |
| PDOX-23 | M | 73 | PDAC | body and tail | 2.0 | MD | Gemcitabine | Y | - | 103 | 69 | Success |
| PDOX-24 | M | 66 | PDAC | body and tail | 4.0 | PD | - | Y | - | 29 | 29 | Failure |
| PDOX-25 | M | 47 | PDAC | body and tail | 3.9 | PD | - | Y | - | 22 | 22 | Success |
| PDOX-26 | M | 62 | PDAC | head | 4.0 | PD | - | Y | - | 96 | 96 | Failure |
| PDOX-27 | M | 67 | PDAC | body and tail | 7.2 | PD | Gemcitabine plus Abraxane | Y | Y | 272 | 176 | Failure |
| PDOX-28 | M | 56 | PDAC | head | 2.6 | PD | FOLFIRINOX | Y | - | 41 | 41 | Success |
| PDOX-29 | M | 62 | PDAC | head | 2.1 | MD | Gemcitabine plus Capecitabine, GV1001 | Y | Y | 235 | 133 | Failure |
| PDOX-30 | F | 49 | PDAC | body and tail | 2.3 | MD | Gemcitabine plus Abraxane | Y | - | 61 | 61 | Failure |
| PDOX-31 | M | 68 | PDAC | body and tail | 4.4 | MD | Gemcitabine plus Abraxane | - | Y | 285 | 285 | Failure |
| PDOX-32 | M | 64 | PDAC | body and tail | 6.9 | PD | Gemcitabine plus Abraxane | Y | Y | 118 | 60 | Success |
| PDOX-33 | F | 42 | PDAC | body and tail | 3.7 | PD | Gemcitabine plus Abraxane | Y | Y | 84 | 84 | Failure |
| PDOX-34 | F | 59 | PDAC | body and tail | 5.5 | PD | Gemcitabine plus Abraxane | - | - | 156 | 151 | Failure |
| PDOX-35 | F | 74 | PDAC | body and tail | 4.0 | PD | - | - | - | 112 | 5 | Success |

(MD, moderately differentiated; PD, poorly differentiated; TTP, time-to-progression)
